# The correlation between 9-HPT and patient-reported measures of upper limb function in multiple sclerosis: a systematic review and meta-analysis

**DOI:** 10.1007/s00415-023-11801-3

**Published:** 2023-06-09

**Authors:** Erica Grange, Claudio Solaro, Rachele Di Giovanni, Davide Marengo

**Affiliations:** 1 CRRF “Mons. Luigi Novarese”, Moncrivello, VC Italy; 2 Scientific Research Area, Italian Multiple Sclerosis Foundation (FISM), Genoa, Italy; 3 Neurology Unit, Galliera Hospital, Genoa, Italy; 4 Department of Psychology, University of Turin, Turin, Italy

**Keywords:** Multiple sclerosis, Upper limb function, 9-Hole Peg Test, Patient-Reported Outcome Measures, Meta-analysis

## Abstract

Upper limb function is one of the most affected domains in people with multiple sclerosis (PwMS), as self-reported by 50% of patients. Heterogeneous results have been found about the correlation between objective and subjective upper limb function. The aim of the present study is to perform a systematic review and meta-analysis of studies presenting data on the strength of association between the gold standard for 9-Hole Peg Test scores and Patient-Reported Outcome Measures (PROMs) of manual ability. Primary research studies including assessments of 9-Hole Peg Test scores and Patient-Reported Outcome Measures were searched in Scopus, Web of Science, and PubMed. Meta analytical calculations were performed using a random-effects model. We retrieved *n* = 27 studies including *n* = 75 distinct effect sizes (*N* of subjects = 3263). The central tendency analysis showed a strong correlation between 9-HPT scores and PROMs (*r* = 0.51, 95% CI [0.44, 0.58]). Moderator analysis showed the effect size to be significantly larger in studies with a mean or median EDSS level indicating severe disability. The publication bias hypothesis was not supported; instead, we noted that studies based on larger samples also tend to report stronger effect sizes. Results of the study indicate that the correlation between 9-HPT and PROMs is strong, although the constructs measured by these instrument does not fully overlap. The correlation between 9-HPT and PROMs was stronger in larger studies and when samples include a sizeable subgroup of PwMS with severe disability, pointing out the importance of sample diversity.

**Supplementary Information:**

The online version contains supplementary material available at 10.1007/s00415-023-11801-3.

## Introduction

Multiple sclerosis (MS) is an autoimmune disease affecting the white and gray matter in the central nervous system, characterized by chronic disease progression and a wide range of neurological symptoms [1]. It occurs most commonly in young adults with higher onset incidence between 20 and 40 years of age, with a double incidence in female sex. MS is characterized by an unpredictable course with a wide range of neurological symptoms [2]. The clinical course of MS is classified according to three main phenotypes: relapsing–remitting (RR), primary progressive (PP), and secondary progressive (SP). The RR phenotype is characterized by episodes of acute worsening of neurologic functioning with total or partial recovery and no apparent progression of the disease. In turn, PP is characterized by steadily worsening neurologic function from the onset of symptoms, without the initial relapses or remissions. Finally, SP is an evolution of the RR phenotype, when the disease becomes more steadily progressive, with or without relapses [3].

Upper limb function (ULF) is one of the most affected domains in people with MS (PwMS), and Holper et al. [4] highlight that 50% of people with MS report self-perceived upper limb dysfunction. Despite this, dysfunction of the upper limbs (UL) has often been considered less debilitating than lower limbs impairment in MS, however, it is associated with a loss of independence in activities of daily living, reduced quality of life, and limitations on participation [5, 6].

As a result of a revision on clinical tools to measure objective ULF in MS, the Nine Hole Peg Test (9-HPT) has been considered the gold standard for UL assessment [7] and one of the best proxies for measuring UL capacity in MS [8]. However, 9-HPT does not assess subjects’ perceived ability in performing manual activity of daily living (ADL) and it is not known the correlation with the level of independence [9].

In the last decade, Patient-Reported Outcome Measures (PROMs) have been introduced in clinical practice and scientific trials [10] to overcome this issue. A recent review reported [8] as the most used PROMs for perceived ULF in MS are the Manual Ability Measure-36 [11] (MAM-36), the ABILHAND [12], and the Disability of Arm, Shoulder and Hand (DASH) [12]. The instruments, although validated in the MS population, were not designed specifically for targeting the MS population. In addition, a new specific PROM for measuring arm function in MS, the Arm Function in Multiple Sclerosis Questionnaire (AMSQ) was developed [13]. These instruments assess perceived ULF in performing ADL by means of multiple self-administered items typically consisting of a description of common unimanual or bimanual tasks (e.g., eating, dressing, buttoning clothes, etc.). In responding to the items, PwMS are required to rate their ability via Likert rating scales. Additionally, in some studies, perceived ULF is assessed using single-item measures or subscales included in larger instruments assessing broader constructs (e.g., perceived quality of life or disability).

Several studies reported the correlation between objective ULF, measured through the 9-HPT, and subjective perception of performing manual activity of daily living (ADL) measured through PROMs; however, heterogeneous correlations were reported. As previously reported, the 9-HPT does not cover the subjects’ perceived ability in performing manual activity of daily living; for this reason, recent studies included also perceived performance measures to investigate the use of upper limb performance during ADL. These measures appear to cover other aspects of upper limb function than the objective UL measures, because the correlations between them vary from low to high [7]. Indeed, previous studies [14, 15] have reported that although scores on objective measures are almost normal, PwMS report upper limb disability affecting their ADL performance.

In light of these considerations, the aim of the present study is to provide an overview of studies presenting data on the strength of association between 9-HPT scores and manual ability as perceived by MS patients. By determining the expected correlation between 9-HPT and PROMs assessing upper limb function in multiple sclerosis, clinicians and researchers can better understand how upper limb function may affect the ability in performing ADL, and thus the overall quality of life of PwMS, that is the ultimate goal of treatment [16]. Additionally, establishing the expected strength of association between the 9-HPT and PROMs helps to validate the use of both assessment tools in evaluating ULF in MS. Demonstrating that these measures are consistent would strengthen their validity, providing a stronger basis for their use for clinical decision-making in a patient-centered care perspective. The lack of strong association between these measures would also support the need of considering both objective and subjective measures to obtain a more nuanced and comprehensive understanding view of patient's challenges in ADL, and tailor and monitor interventions accordingly [7].

For this purpose, we plan to review the existing literature and conduct a meta-analysis to synthesize the central tendency and heterogeneity of the correlations between 9-HPT and ULF PROMs documented by published studies, as well as providing evidences on possible publication bias affecting the current literature. Finally, we aim to determine whether different characteristics of the selected studies, including the sample demographic and clinical characteristics, and coding of 9-HPT, can help explaining the heterogeneity of correlations between the 9-HPT and ULF PROMs.

## Materials and methods

We conducted a systematic review of the literature and a meta-analysis of studies presenting results on the correlation between 9-HPT and ULF PROMs in MS, following the Preferred Reporting Items for Systematic Reviews and Meta-Analyses (PRISMA) guidelines [17]. The meta-analysis was registered in the international prospective register of systematic reviews (PROSPERO 2021 CRD42021289036).

### Eligibility criteria

We aimed to include all study designs of quantitative primary research that involved assessments with 9-HPT and an ULF PROM in people with MS, reporting correlation between the measures of their scores. In selecting the studies, we employed the following exclusion criteria: (1) studies with either objective outcome measures or PROMs alone, but not both; (2) studies with mixed populations; (3) non-peer‐reviewed publications. No limitations were put on date or language of publication.

### Database searches

To retrieve documents, we searched the following citation databases: Scopus, Web of Science, and PubMed. Reference lists reported in retrieved documents were checked to find additional potential eligible studies. Finally, where needed, we contacted the experts in the field. The following strategy was used to search Scopus: we generated a search query searching the *title*, *abstrac*t, and *keywords* record fields using two groups of keywords combined with an AND statement. The first group of keywords was intended to detect papers presenting results on sample of subjects with a MS diagnosis (i.e., *multiple sclerosis*, *ms*, *spms*, *rrms*, and *ppms*), while a second group of keywords were intended to detect papers including data on the 9-HPT (i.e., *9HPT*, *9-HPT*, *nine hole peg test*, *nhpt*, and *9-Hole Peg Test*).

This query strategy was then adapted to generate the query string for use in the Web of Science and PubMed databases. The searches were conducted between December 2021 and June 2022. The used queries are reported in full in the Supplementary material.

### Study selection process

Study selection was performed through Rayyan software independently by two authors. First, we inspected all records for duplicates identified by searching the databases. Next, the remaining records were screened for eligibility according to inclusion and exclusion criteria based on information reported in the title, abstract, and the full text of the manuscript. In case of disagreement in the articles selection, it was discussed by the two authors.

### Data extraction

All selected papers were inspected for information including the year of publication of the paper, demographic and clinical information, the procedure use to perform the assessment of ULF, and effect sizes representing the association between 9-HPT and PROMs. Data were extracted from selected papers based on a predetermined coding sheet.

Regarding demographic information, the following information was retrieved about sample characteristic: distribution of age of (mean or median) and gender (number/percentage of PwMS by gender group). Clinical information about the sample extracted from the paper included information about distribution of MS disease course (number of PwMS per disease course), and disease duration (mean/median). Additionally, we collected information on the distribution of clinician-rated Expanded Disability Status Scale (EDSS; mean or median). The EDSS is a widely used ordinal measure quantifying disability and disease progression in subjects with multiple sclerosis (Kurtzke in 1983 [18]). EDSS scores derive from neurological examinations and range from 0 (normal neurological exam) to 10 (death due to MS) [18].

We also extracted information about the assessed ULF measures. We retrieved information about the scoring of 9-HPT, including whether the test was scored based on a single or both arms, and scoring metric (e.g., seconds); regarding the administered PROMs, including bibliographic information about the instrument, as well as the number of items included in the assessment.

Next, we extracted effect sizes representing the correlation between 9-HPT and PROMs. First, we retrieved information about the type of correlation used to evaluate the association between the 9-HPT and PROM scores (Pearson vs. Spearman). Note that PROMs may be scored either to indicate ULF, or reverse scored to indicate lack of ULF. In a similar way, the 9-HPT may be scored to reflect inability to perform the task (i.e., seconds required to complete the task) or ability (e.g., pegs per second). Depending on the scoring strategy used, a positive correlation may thus indicate convergence or divergence between the two measures in assessing ULF. For the purpose of the present study, when necessary, all correlations were recoded as positive when the reported correlation indicated convergence between the two measures, and recoded as negative when the correlation indicated a divergence between the measures. When we could not find correlations reported in the included studies (e.g., correlations were either not reported in full, or other effect sizes were reported but available information could not be used to obtain correlations), we contacted the first or corresponding authors asking them to provide us with missing correlations (i.e., authors were asked to compute correlations and provide us with the results). Study authors were contact by email five times over the course of 2 months.

### Study quality assessment

Quality of the included studies was assessed by two independent critical appraisers (EG and RDG) using an adapted version of the Joanna Briggs Institute Critical Appraisal tools for cross‐sectional studies. More specifically, the appraisers scored each paper based on the following criteria: (1) Clear definition of subject inclusion criteria; (2) Detailed description of study subjects; (3) Use of valid and reliable measures for the assessment of the study outcomes; and the (4) Appropriateness of statistical analyses. For each criterion, appraisers rated “Yes” if the criterion was fully respected, “No” if not respected at all, “Unclear” if the criterion was partially respected, or applicability was uncertain. Each item was scored 1 for “Yes” responses, and 0 for either “No” or “Unclear” responses. Any disagreement was discussed and resolved by the two critical appraisers.

### Data analysis

We use correlations (Pearson or Spearman) to express the association between 9-HPT scores and PROM of ULF. Following the indications by Schmidt and Hunter (2014), collected effect sizes were not transformed into Fisher's *z* scores, since this conversion is not indicated for meta-analytic random-effects models; they yield an upward bias in the estimation of mean correlation, which is normally higher than the bias due to the usage of untransformed correlations. The meta-analysis was performed using a random-effects model as the true effect size was likely to vary in the individual studies, owing to the variety in data sources, study designs, and analytic approaches.

Because most of the studies provided more than one effect size computed on the same sample at one or more time points, thus resulting in a lack of independence of among the retrieved effect sizes within the same study, in estimating the meta-analytical correlation, we used a multilevel approach. More specifically, we implemented three-level meta-analytic model modeling three different variance components: sampling variance of the extracted effect sizes (i.e., the indeterminacy in effect sizes due to the use of samples, as opposed to population data to compute effect sizes); variance at the effect size level (i.e., within-cluster variance); variance at the study level (i.e., between-cluster variance). Note that in interpreting the magnitude of the meta-analytical correlation, we refer to the existing guidelines indicating correlations equal or above *r* =|0.10|, *r* =|0.30|, and *r* =|0.50| as reflecting, respectively, small, media, and strong effect sizes [19]. Heterogeneity of effect sizes was investigated by computing the *Q* test of heterogeneity, the *I*^2^ statistic representing the proportion of true variation in observed effects, and by determining the percentage of heterogeneity due to the different variance components [20]. Note that Grubb's test was used to identify outliers prior to meta-analytical computations. Publication bias was investigated by inspecting the funnel plot of studies’ effect sizes against their relative standard error. Symmetry of the funnel plot was determined using a modified Egger’s intercept test [21]. More specifically, we fitted a multilevel model predicting study effect sizes with sampling standard errors (i.e., the square root of sampling variance) as a moderator: significance of the moderator effect would indicate a significant association between the standard error and effect size, indicating a potential “small-study” effect biasing our results. Classic fail-safe N was then used to evaluate the impact of a file-drawer problem (e.g., the number of unpublished studies reporting non-significant associations that would nullify emerging meta-analytical associations).

To determine the source of heterogeneity in effect sizes, we performed a series meta-regression analyses. First, we investigated whether the use of specific PROMs had an impact on the correlation with 9-HPT scores: to reach this aim, contrasts between specific PROMs were investigated using dummy coding. Note, however, that these contrasts were only examined if at least four non-independent studies per PROM were available [22]. Additionally, meta-regressions were performed separately for following variables: length of PRO questionnaire (i.e., number of items), type of correlation (Pearson = 1; Spearman = 0), coding of 9-HPT scores (seconds = 1; else = 0), source of 9-HPT score (Total score = 1; Single arm = 1), year of publication, and the following sample characteristics: mean/median age, prevalence of gender (% of female patients), disease course (% of RR patients), and overall severity of disability in the sample (EDSS ≥ 6.0 vs lower EDSS). Finally, we checked for the impact of study quality, using a median split on the overall quality score approach to distinguishing between high-to-moderate vs low quality (Quality score ≥ median = 1; else = 0). All analyses and visualization of results were performed using the *metafor* package for R [23, 24].

## Results

### Study selection

**Fig. 1:**
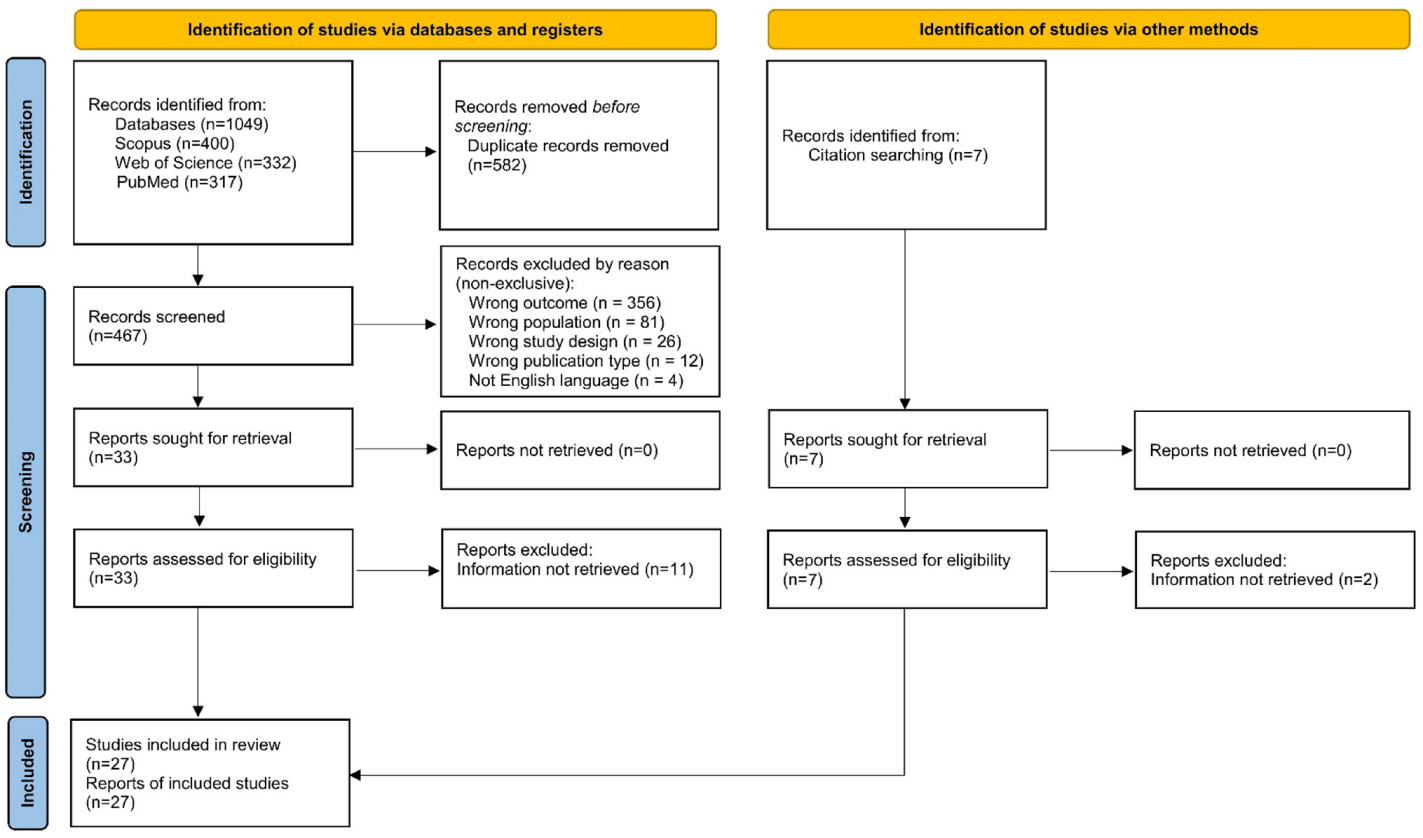
Literature search and study selection according to PRISMA 2020 flow diagram

The PRISMA diagram in Fig. 1 provides a description of study selection flow. A total of *n* = 1049 records were retrieved by querying the databases (Scopus: *n* = 400; Web of Science: *n* = 332; PubMed: *n* = 317). First, we inspected all records for duplicates identified by searching the databases, resulting in the removal of *n* = 582 records. The remaining records (*n* = 467) were screened based on the information reported in the title, abstract, and the full text of the manuscript. This step led to identification of *n* = 22 eligible studies by both authors; while n = 18 studies were selected by only one of the authors and after disagreement discussion, they result in the further inclusion of *n* = 11 studies, leading to a total of 33 records. Additionally, *n* = 7 studies were included based on the inspection of study references [25–31]. Hence, a total of n = 33 eligible studies were identified.

There were n = 21 [30–50] studies that did not include information about the correlation between 9HTP and a validated PROM, but only their scores. After contacting the authors, missing information could be retrieved for *n* = 8 studies [32–39]. Finally, we include in the review and meta-analysis a total of *n* = 27 studies [14, 25–29, 32–39, 51–63].

### Overview of selected studies

The characteristics of included studied are reported in Table 1. The included studies involved a total of 3263 subjects, with a mean (or median) age ranging from 37.60 [26] to 58.2 [14], and generally a prevalence of female subjects, with an average of 66.8% females ranging from 50.0% [14] to 84.8% of the sample [62]. As regards disease course, RR patients were on average 63.4% of the sample, ranging from 0% [14, 39] to 100% of the sample [36, 58]; in turn, patients with either primary or secondary progressive MS were on average 36.6% of the samples. Please note that some of the studies did not report information about the distribution of disease course in the sample, and thus were excluded from these calculations (*n* = 4) [26, 32, 51, 52]. As regards EDSS, 16.0% of the studies reported either median or mean EDSS values ≤ 0.2.5, 60.0% of the studies reported either median or mean EDSS values in the 3.0–5.5 range, and 24.0% of the studies reported either median or mean EDSS values ≥ 6.0. Please note that *n* = 2 studies did not include information about the distribution of the EDSS in the sample [26, 32].

**Table 1:** Characteristics of studies included in the meta-analysis

	*n*^a^	Age	Gender	Disease duration	Disease course	EDSS	PROM (n. items)	9HPT Scoring
Afshar et al. (2021) [62]	155	40.78 ± 0.88	123F/22 M	–	95RR/30SP/24PP	Mean ± DS 2.91 ± 1.38 women 4.04 ± 1.45 men	AMSQ (31)	s
Boffa et al. (2020) [39]	26	52 ± 13 Exp 57 ± 7 Ctrl	14F/12 M	19 ± 10 Exp; 13 ± 12 Ctrl	17SP/9PP	Median [range] 6.0 [4.0–7.5]	ABILHAND (23)	s
Cetisli Korkmaz et al. (2018)36	49	40.33 ± 10.22	38F/11 M	5.96 ± 4.38	49RR	Mean ± SD 1.88 ± 1.65	DHI (18)	s
Ertekin et al. (2020) [29]	200	38.8 ± 10.8	139F/61 M	–	175RR/22SP/3PP	2.4 ± 2.1	MAM-36 (36)	s
Gandolfi et al. (2018) [35]	39	51.96 ± 10.87 Exp group 50.67 ± 10.80 Ctr group	–	13.48 ± 7.82 Expt group 14.19 ± 9.78 Ctrl group	26RR/15SP/3PP	Median [Q1-Q3] 6.00 [5.00–6.60] Expt group 6.00 [4.00–7.25] Ctrl group	MAL (30)	s, peg/s
Gatti et al. (2015) [32]	19	46 ± 9.6	12F/7M	–	SP/PP	–	ABILHAND (23)	s
Gold et al. (2003) [25]	187	39.4 ± 9.8 No cognitive impaired group 42.9 ± 9.4 Definite cognitive impairment group	117 F/70M	8.6 ± 8.9 No cognitive impaired group 11.4 ± 8.2 Definite cognitive impairment group	75RR/53SP/29PP 17Not defined 13First year of diagnosis	Mean ± SD 2.3 ± 1.5 No cognitive impaired group 5.1 ± 1.8 cog Definite cognitive impairment group	HAQUAMS -Mobility upper limb score (5)	s
Grange et al. (2021) [28]	243	48.95 ± 14.51	159F/86F	14.02 ± 10.87	154RR/59SP/32PP	Mean ± SD 4.41 ± 2.12	ABILHAND-26 (26)	s
Healy et al. (2019) [58]	364	49.9 ± 10.7	263F/94M	17.3 ± 10.3	303RR/61Progressive	Median [range] 2 [0–7.5]	CAT version of the NeuroQOL UE (≥ 8)	*z*-score (s)
Heldner et al. (2014) [55]	42	47.8 ± 12.2	63F/38M	12.2 ± 9.9	61RR/30SP/8PP/2CIS	Mean ± SD 3.7 ± 1.8	Adapted Dexterity Questionnaire Sunderland (24)	s
Huertas-Hoyas et al. (2020) [61]	30	45 ± 8.11	16F/14M	8.8 ± 4.84 (RR) 5.4 ± 4.60 (PP) 16.78 ± 7.77 (SP)	15RR/7SP/8PP	Mean ± SD 4.2 ± 1.99 RR 6.1 ± 0.62 PP 5.5 ± 1.02 SP	ABILHAND (23)	s
Kamm et al. (2015) [33]	39	49.20 ± 10.87 dexterity group 51.89 ± 8.02 theraband group	26F/13M	15.38 ± 9.95 dexterity group, 15.95 ± 9.68 theraband group	27RR/11SP/1PP	Mean ± SD 4.28 ± 1.48 dexterity group 4.82 ± 1.04 theraband group	Adapted Dexterity Questionnaire Sunderland (24)	s
Lamers et al. (2013) [14]	30	58.2 ± 10.9	15F/15M	21.8 ± 11	25SP/5PP	Median [IQR] 7.5 [7–8]	MAL (30)	peg/s
Lamers et al. (2015) [56]	105	53.7 ± 11.1	62F/43M	17.93 ± 11.18	34RR/58SP/13PP	Median [Q1–Q3] 6.5 [5.1–7.5]	MAM-36 (36)	peg/s
Marrie et al. (2011) [53]	44	42.2 ± 8.1	35F/9M	8.3 ± 6.7	30RR/10SP/1PP/1CIS 2unknown	Median [IQR] 3.5 [2–4]	MSPS—Hand Score (1)	*z*-score (s)
Mate et al. (2019) [37]	188	42.6 ± 9.7 male 44 ± 11.6 female	140F/48M	6.1 ± 3.4 male 6.6 ± 3.9 female	98RR/7SP/3PP/9CIS	median [IQR] 2 [1–3] women 2 [1–5] men	DASH (20)	peg/s
Molenaar et al. (2022) [63]	533	50.85 ± 12.39	333F/200 M	13.41 ± 8.20	299RR/132SP/102PP	Median (range) 4.0 (0.0–8.5)	AMSQ (31)	s
Ozdogar et al. (2020) [38]	59	40.1 ± 10.7	43F/16M	6.6 ± 4.8	54RR/5SP	mean ± SD 2.4 ± 1.4	MAM-36 (36)	s
Padua et al. (2007) [26]	80	37.6 (–)	58F/22M	–	–	–	DASH (20	–
Rossier et al. (2002) [51]	43	53.8 ± 10.3	29F/14M	19.2 ± 10.8	–	Mean ± SD 7.3 ± 1.5	GNDS—Arm score (6)	s/peg
Rudick et al. (2014) [54]	51	46.2 ± 10.1	40F/11M	–	35RR/13SP/2PP/1CIS	Mean ± SD 3.9 ± 1.8	MSPS—Hand Score (1)	s
Savin et al. (2016) [34]	26	48.42 ± 9.86	16F/10M	11.91 ± 7.48	22RR/1PP/3RP	Median [range] 4.75 [2.0–6.0]	ABILHAND (23)	s
Solaro et al. (2020) [60]	218	48.06 ± 14.39	145F/73M	13.92 ± 10.47; median [range] 11 [0–53]	141RR/53SP/24PP	Mean ± SD 4.19 ± 2.11	MAM-36 (36)	s
Steinheimer et al. (2018) [57]	100	40.67 ± 10.72	69F/31M	10.45 ± 8.44 [range, 0.5–40]	87RR/8SP/1PP/4CIS	Mean ± SD 3.6 ± 2.0	AMSQ (31)	s
van Leeuwen et al. (2017) [27]	105	47.6 ± 11.6	61F/51M	Median [range] 13 [1–49]	57RR/24SP/13PP/18CIS	Mean ± SD 5.4 ± 2.7 self-administered	AMSQ (31)	s
van Munster et al. (2019) [59]	257	46.6 ± 12.8	171F/86M	14.9 ± 11.7	186RR/45SP/15PP/11CIS	Median [IQR] 3.0 [2]	AMSQ (31)	s
Yozbatiran et al. (2006) [52]	31	39.74 ± 10.10	25F/6M	–	–	Mean ± SD 2.56 ± 1.91	UEI (1)	*z*-score (s)

^a^Sample size used for the meta-analytic computations

Concerning the studied outcomes, in most of the studies, the 9-HPT was only scored by recording the number of seconds required to move the pegs (*n* = 26) [25–29, 32–36, 38, 39, 51–54, 57–63], while a minority of studies used an alternative scoring based on the peg per second ratio (*n* = 3) [14, 37, 56]. Please note that we could only find one study using both these coding procedures [35].

Thirteen different ULF PROMS have been used among the included studies. Some of the PROMs assess upper limb ability in performing ADL, namely the ABILHAND-23 (n = 4) [32, 34, 39, 61], ABILHAND-26 (*n* = 1) [28], Manual Dexterity in Multiple Sclerosis adapted from Sunderland [64] (*n* = 2) [33, 55]), MAL (*n* = 2) [14–35, MAM-36 (*n* = 4) [29, 38, 56, 60], and NeuroQOL UE (n = 1) [58]. Other PROMs assess UL disability, namely the AMSQ (*n* = 5 studies) [27, 57, 59, 62, 63], DASH (*n* = 2) [26, 37], Duruoz's Hand Index [36], Upper extremity index (*n* = 1) [52], HAQUAMS’ Upper Mobility Subscale (*n* = 1) [25], Guy’s Neurological Disability Scale-Arms score (*n* = 1) [51], and the Performance scale-Hand Score (*n* = 2) [53, 54]). Finally, it is important to highlight that several PROMs are not validated for the MS population [33, 36, 52, 55], and others consist of either single items or multi-item scales included in PROMs assessing additional constructs beyond upper limb function [25, 51, 53, 54].

### Quality of included studies

Results of evaluation of the quality of studies are reported in Table S1 of the Supplementary Material. Only n = 2 study [29, 35] reported the maximum score of 4, n = 4 studies [28, 37, 38, 59] reported a score of 3, n = 9 studies 14, [33, 39, 50, 53, 56, 57, 60, 63] reported a score of 2, *n* = 11 studies [25–27, 32, 34, 36, 54, 55, 58, 61, 62] reported a score of 1, and *n* = 1 study [52] reported a score of 0. At a median value of 2 (Range 0–4), study quality was generally low-to-moderate. Overall, the majority of the selected studies reported adequate inclusion criteria (*n* = 18; 67%) and reliability and validity of UL measures (*n* = 16; 59%), while only a minority of studies provided in-depth information about study subjects and setting (*n* = 6; 22%) and rationale for using specific statistical analyses (*n* = 8; 30%). In particular, as regards the last criterion (i.e., adequacy of statistical analyses), most of the studies failed to report and/or discuss information on the distributional characteristics of study measures.

### Central tendency and heterogeneity

**Fig. 2:**
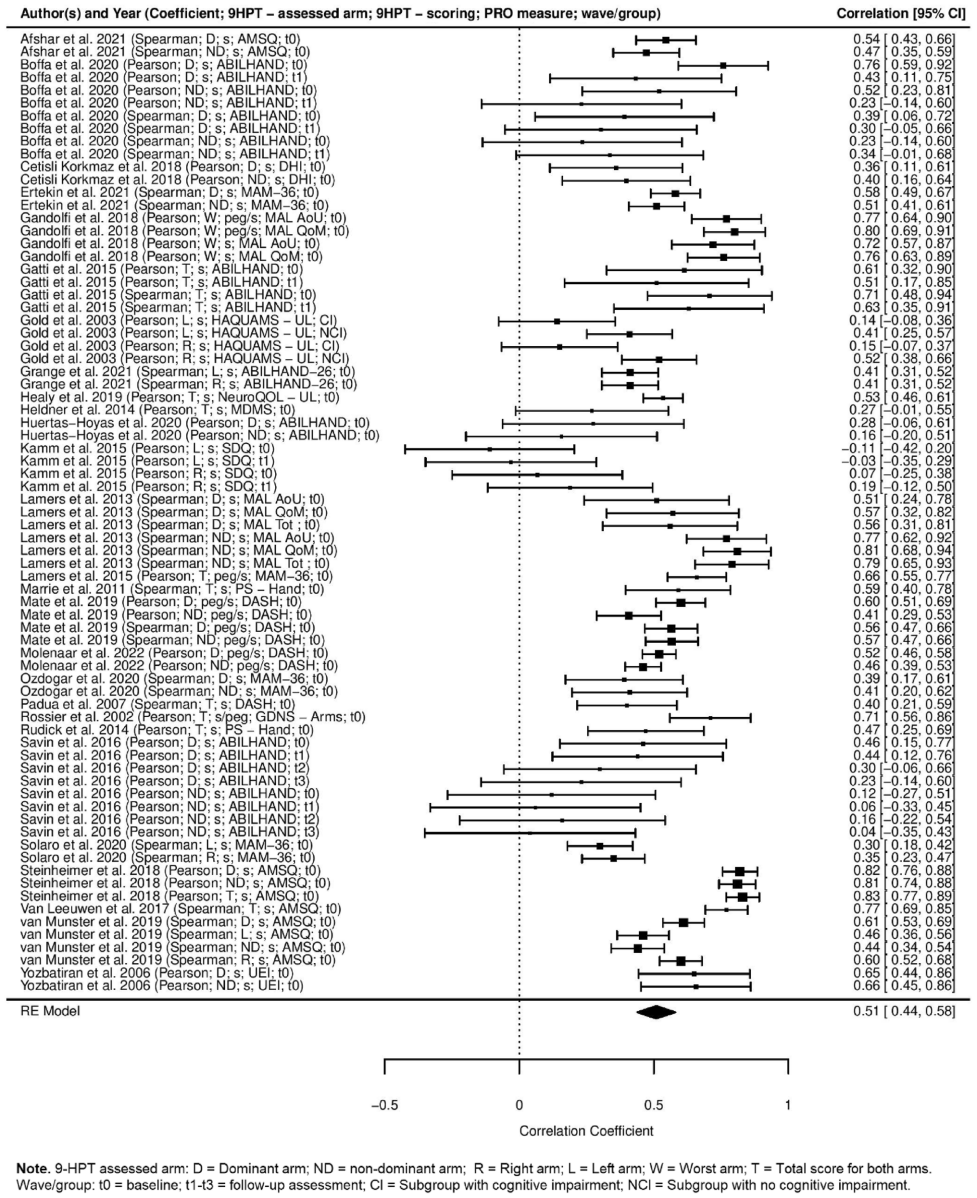
Forest plot of effect sizes of the association between 9-HPT scores and PROMs assessing upper limb function

A forest plot of study effect sizes representing the correlation between 9-HPT scores and ULF PROM scores are shown in Fig. 2. Overall, we examined *n* = 75 distinct effect sizes reported in *n* = 27 studies. Grubbs test failed to identify outliers prior to meta-analytical computations. Overall, the central tendency analysis showed a strong association between 9-HPT scores and ULF PROMs scores (*r* = 0.51, 95% CI [0.44, 0.58]). The Q test for heterogeneity was significant (*Q* (74) = 515.68, *p* < 0.0001), indicating the presence of non-negligible heterogeneity among the effect sizes, but observed dispersion of effect sizes was mostly due to true heterogeneity (*I*^2^ = 87.55). In particular, based on model decomposition of effect size variance, we saw that for all traits, most of the heterogeneity was due to variance at the study level (80.79%, between-cluster variance), followed by sampling variance (12.45%, variance due to sampling error), while variance at effect size level was the lowest (6.76%, within-cluster variance). Note that Grubb's test failed to identify outliers among the collected effect sizes.

### Publication bias

**Fig. 3:**
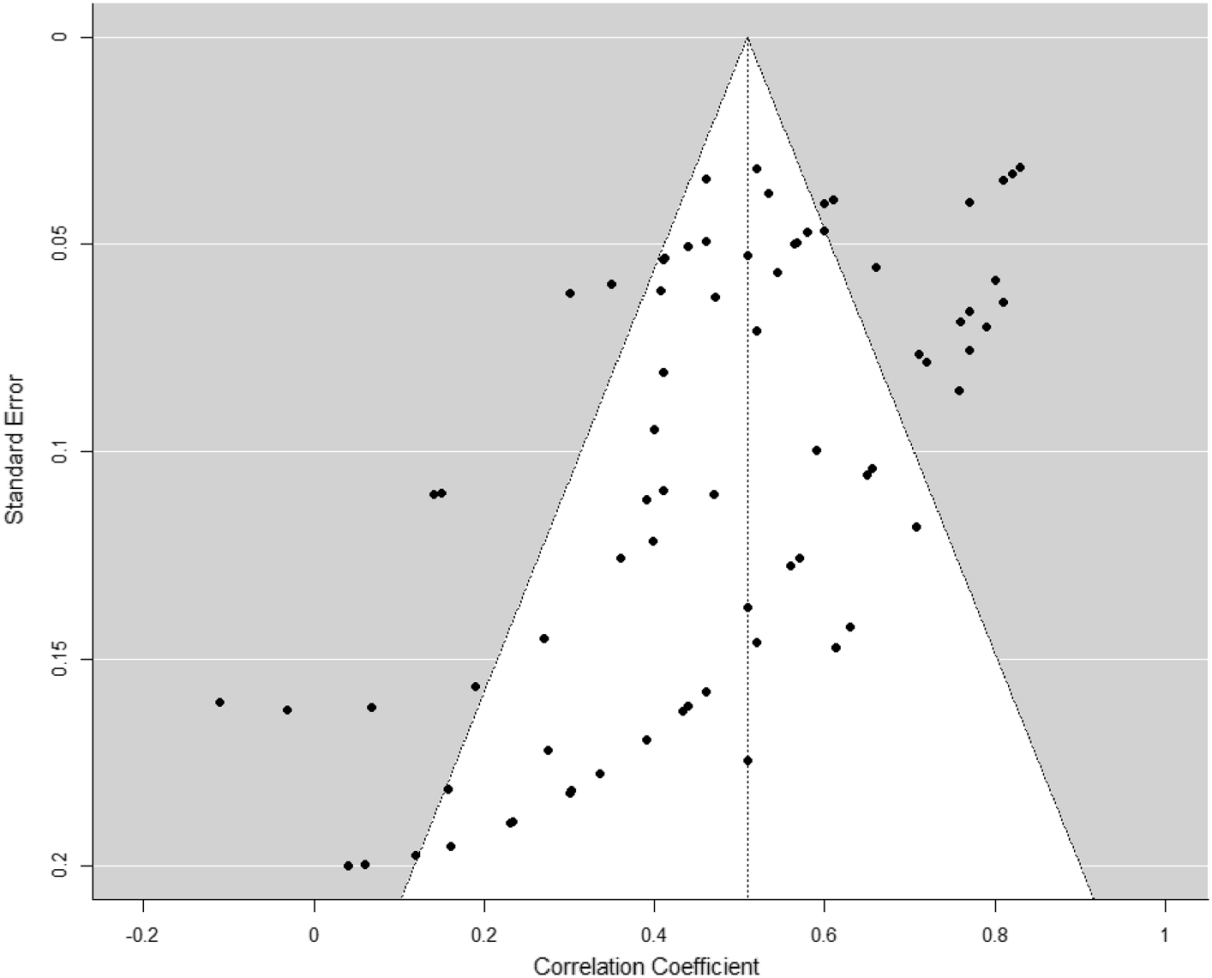
Funnel plot of effect sizes against their standard error

The funnel plot of standard errors versus the study correlations was markedly asymmetric (see Fig. 3). However, contrary to the publication bias assumption (a positive correlation between effect size and its standard error), Egger’s tests was significant but a negative association was found between standard errors and correlations (*b* (73) = − 4.02 [− 5.10, − 2.94], p < 0.001), indicating that studies based on larger samples also tended to report stronger effect sizes. Additionally, the computed fail-safe *N* of 99,190 value was significantly larger than the recommended rule-of-thumb limit (5 × number of effect sizes + 10 = 385) [65]. These findings support the significance of the meta-analytic correlation emerging for the trait, ruling out the existence of a relevant publication bias problem.

### Moderator analyses

Finally, we look at the results of moderator analyses. Estimated effects are reported in Table 2. A significant effect was found indicating a larger effect size for the association between PROM and 9-HPT when using the AMSQ questionnaire as opposed to the ABILHAND questionnaire (*β* = 0.212, *p* = 0.044). Note that contrasts between PROMs could only be examined between the AMSQ, MAM-36, and ABILHAND questionnaires due to the low number of studies identified for the other PROMs (*n* < 4). Finally, we found the effect size to be significantly larger (*β* = 0.186, *p* = 0.026) in studies with a mean or median EDSS level indicating severe disability (EDSS ≥ 6.0) compared with studies performed on samples with lower mean/median disability. No other significant moderation effect emerged.

**Table 2:** Moderator of association between 9-HPT and patient-reported measures of upper limb functionality

	Effect	SE	*t*	*df*	*p*	LL	UL
Used PROM: AMSQ vs. MAM-36	0.142	0.104	1.362	17	0.191	– 0.078	0.362
Used PROM: AMSQ vs. ABILHAND	0.212	0.101	2.097	34	0.044	0.007	0.418
Used PROM: MAM-36 vs. ABILHAND	0.070	0.104	0.670	29	0.508	– 0.143	0.282
Number of items in PROM	0.000	0.003	0.086	73	0.931	– 0.005	0.006
Scoring of 9-HPT (Seconds = 1; else = 0)	– 0.081	0.070	– 1.149	73	0.254	– 0.220	0.059
Source of 9-HPT score (Total score = 1; Single arm = 0)	0.067	0.055	1.234	73	0.221	– 0.042	0.176
Type of correlation (Pearson = 1; Spearman = 0)	– 0.002	0.046	0.038	73	0.970	– 0.094	0.090
Year of publication	– 0.003	0.007	– 0.457	73	0.649	– 0.017	0.010
Age (sample mean/median age)	0.004	0.007	0.615	73	0.541	– 0.009	0.017
Gender (% of female patients)	– 0.002	0.004	– 0.490	73	0.626	– 0.010	0.006
Disease course (% of RR patients)	– 0.001	0.001	– 0.854	65	0.396	– 0.004	0.001
Disability (mean/median EDSS ≥ 6.0 = 1; else = 0)	0.186	0.082	2.272	68	0.026	0.023	0.349
Quality of studies (quality score ≥ 2 = 1; else = 0)	0.075	0.073	1.028	73	0.307	– 0.071	0.221

## Discussion

The present study aimed to provide an overview of studies presenting data on the strength of association between 9-HPT scores and manual ability as perceived by MS patients, and to estimate of the central tendency and heterogeneity of the correlations between 9-HPT and ULF PROMs documented by published studies. To our knowledge, our study is the first to provide both a qualitative overview and quantitative analysis of studies reporting on the association between 9-HPT and ULF PROMs in MS patients.

Overall, the meta-analysis showed the existence of a strong correlation between 9-HPT and ULF PROMs (*r* = 0.51, 95% CI [0.44, 0.58]), although a significant heterogeneity was found among the effect sizes included in published studies. For this reason, we examined different characteristics of the selected studies as possible sources of heterogeneity of correlations between the 9-HPT and ULF PROMs. Moderator analyses provided interesting results, suggesting that the correlation between 9-HPT and PROMs may be affected by the specific PROM used. More specifically, in our study, we found that the AMSQ questionnaire showed a higher correlation with 9-HPT scores than the ABILHAND questionnaire, while no differences were found when comparing these two PROMs with the MAM-36. Note that remaining PROMs could not be examined in detail due to the low number of studies reporting their use. Additionally, it is important to highlight that some of the PROMs used in selected studies were not validated for the MS population [33, 36, 52, 55], while other consisted of either single items or multi-item ULF PROMs included in instrument that assesses multiple constructs [25, 51, 53, 54]. Interestingly, the number of items included in the PROMs did not seem to affect the correlation between the 9-HPT and ULF PROMs.

Findings of moderator analyses also pointed toward the clinical characteristics of recruited samples as a source of heterogeneity in effect sizes. More in details, we found that the strength of the association between 9-HPT and PROMs was significantly larger in studies with a mean or median EDSS level indicating severe disability (EDSS ≥ 6.0) when compared with studies performed in samples with overall lower disability. Other characteristics of the selected studies, such as sample size, mean age, percentage of female and male, disease course, as well as heterogeneity on the scoring of 9-HPT, failed to show a significant effect on the association between 9-HPT and ULF PROMs.

The present study also aimed at investing potential publication bias in the selected literature. Our analyses did not support the publication bias hypothesis (i.e., studies reporting stronger effect sizes being more likely to be published than studies with non-significant or negligible effects). Instead, we found evidence that the reported association between 9-HPT and ULF PROMs tend to be stronger in studies recruiting larger samples. A possible interpretation of this effect is related to the increased variability of study measures scores (including ULF measures) in larger samples compared with smaller ones, which is a factor known to affect the strength of correlation (i.e., larger variability is associated with stronger effect sizes) [66].

Note that, on the inspection of the current literature, there appears a general lack of clinical information about recruited samples (e.g., disease course and duration) and setting of assessment reported in published papers. Another limitation of current literature is related to the lack of information on the distributional characteristics of both 9-HPT and PROMs, which is a key factor influencing the decision to use specific statistical procedures to analyze the date (e.g., type of correlation) [67], possibly compromising the validity of emerging findings. On the other hand, it is worthy to note that we could not find evidence that the use of either Spearman or Pearson correlation coefficient significantly affected the size of emerging meta-analytical correlations between 9-HPT and PROMs.

The findings emerging from the present study should be understood in light of some limitations. These include heterogeneity of effect sizes, indicating that the strength of the correlation between 9-HPT and ULF PROMs varied across different studies. Many potential sources of this heterogeneity were explored through moderator analyses, but there may still be unaccounted factors contributing to the variability. Additionally, the limited number of studies for certain PROMs hindered us from and in-depth investigation of the role of specific PROMs in influencing the heterogeneity of correlations observed between 9-HPT and perceived ULF in performing ADL. By examining a larger literature, future review studies might be able to address these limitations to enhance our understanding of the relationship between 9-HPT and PROMs in assessing ULF in multiple sclerosis patients.

In sum, the overall correlation found through the meta-analysis highlighted the existence strong overlap exists between the 9-HPT and PROMs in assessing ULF function, albeit these two kinds of measures are likely to assess different domains of ULF. The lack of a strong convergence is in part expected as the objective assessment provided by the 9-HPT task is influenced by several neurological functions, including coordination and strength, while PROMs provide an assessment of ULF that is necessarily influenced by patients’ expectations, and self-awareness of personal deficits, and availability of personal experiences in performing a variety of ADL [8]. Consequently, an objective assessment of ULF may not sufficient to assess the effectiveness of rehabilitation program or pharmacological treatment. As suggested in the previous studies [7, 16], there is the need to include both clinical and self-reported upper limb outcome measures in clinical trial, to assess the effective benefit of treatment on the ability of performing specific upper limb tasks and level of autonomy, that is the ultimate goal for both researcher and clinician.

### Conclusion

On average, a strong correlation exists between 9-HPT scores and PROMs assessing ULF in ADL of patients with MS, supporting concurrent validity of both measures. However, the correlation does not come close to that expected for establishing equivalence of assessed constructs (e.g., *r* ≥ 0.8, [68]), thus indicating the two forms of measurement indeed assess different constructs. Results of the present study suggest that some questionnaire (i.e., AMSQ) may show a stronger convergence with 9-HPT scores than other instruments (i.e., ABILHAND), although these results should be taken with cautions due to the low number of studies included in the analysis. Finally, the size and the average disability of the recruited sample were found to affect the size of association between 9-HPT and ULF PROMs, such that the association tend to be stronger in large samples, and in those samples including larger groups of patients with severe disability along with less disabled patients.

## Supplementary Information

Below is the link to the electronic supplementary material.

Supplementary file1 (CSV 14 KB)

Supplementary file2 (DOCX 16 KB)

## Data Availability

Study data are provided as supplementary material.
