# Supplementary material for: The correlation between 9-HPT and patient-reported measures of upper limb function in multiple sclerosis: a systematic review and meta-analysis
Source: J Neurol. 2023 Jun 9;270(9):4179–91. doi: 10.1007/s00415-023-11801-3 (PMC10421783; doi:10.1007/s00415-023-11801-3)
Supplement: Supplementary file 2 — Supplementary file2 (DOCX 16 KB) [file 415_2023_11801_MOESM2_ESM.docx]

**SUPPLEMENTARY MATERIAL**

**Queries used in database searches**

**SCOPUS**

TITLE-ABS-KEYes ( "Multiple Sclerosis" OR ms OR spms OR rrms OR ppms ) ANoD TITLE-ABS-KEYes ( 9hpt OR "9-HPT" OR "NoINoE HOLE PEG TEST" OR nhpt OR "9-Hole Peg Test" )

**MEDLINoE via PUBMED**

(((((("multiple sclerosis"[MeSH Terms]) ) OR (MS)) OR (SPMS)) OR (PPMS)) OR (RRMS)) ANoD (((((9HPT) OR (NoHPT)) OR (9-HPT)) OR (Noine Hole Peg Test)) OR (9-Hole Peg Test))

OVID

**WEB OF SCIENoCE**

ALL=( "Multiple Sclerosis" OR ms OR spms OR rrms OR ppms) ANoD ALL=(9hpt OR "9-HPT" OR "NoINoE HOLE PEG TEST" OR nhpt OR "9-Hole Peg Test")

**Table S1. Quality of studies included in the meta-analysis**

|  | 1.Inclusion  criteria | 2. Description  of study subjects | 3. Validity and reliability  of measures | 4.Statistical  analyses | Score |
| --- | --- | --- | --- | --- | --- |
| **Afshar et al. 2021 ^51^** | Unclear | Unclear | Yes | Unclear | 1 |
| **Boffa et al. 2020^27^** | Yes | Unclear | Yes | No | 2 |
| **Cetisli Korkmaz et al. 2018^24^** | Yes | Unclear | Unclear | Unclear | 1 |
| **Ertekin et al. 2021** | Yes | Yes | Yes | Yes | 4 |
| **Gandolfi et al. 2018^23^** | Yes | Yes | Yes | Yes | 4 |
| **Gatti et al. 2015^20^** | Yes | Unclear | Unclear | Unclear | 1 |
| **Gold et al. 2003^16^** | Unclear | Unclear | Yes | Unclear | 1 |
| **Grange et al. 2021^19^** | Yes | Unclear | Yes | Yes | 3 |
| **Healy et al. 2019^47^** | No | Yes | Unclear | Unclear | 1 |
| **Heldner et al. 2014^44^** | Yes | Unclear | Unclear | Unclear | 1 |
| **Huertas-Hoyas et al. 2020^50^** | Yes | Unclear | Unclear | Yes | 1 |
| **Kamm et al. 2015^21^** | Yes | Yes | No | Unclear | 2 |
| **Lamers et al. 2013^42^** | Yes | Unclear | Unclear | Yes | 2 |
| **Lamers et al. 2015^45^** | Yes | Unclear | Yes | Unclear | 2 |
| **Marrie et al. 2011^41^** | Yes | Unclear | Unclear | Yes | 2 |
| **Mate et al. 2019^25^** | Unclear | Unclear | Yes | Unclear | 3 |
| **Molenaar et al. 2022^52^** | Unclear | Yes | Yes | Unclear | 2 |
| **Ozdogar et al. 2020^26^** | Yes | Unclear | Yes | Yes | 3 |
| **Padua et al. 2007^17^** | Unclear | Unclear | Yes | Unclear | 1 |
| **Rossier et al. 2002^39^** | Yes | Unclear | Yes | Unclear | 2 |
| **Rudick et al. 2014^43^** | Yes | Unclear | Unclear | Unclear | 1 |
| **Savin et al. 2016^22^** | Unclear | Unclear | Yes | Unclear | 1 |
| **Solaro et al. 2020^49^** | Yes | Unclear | Yes | Unclear | 2 |
| **Steinheimer et al. 2018^46^** | No | Yes | Yes | Unclear | 2 |
| **van Leeuwen et al. 2017^18^** | Yes | Unclear | Unclear | Unclear | 1 |
| **van Munster et al. 2019^48^** | Yes | Unclear | Yes | Yes | 3 |
| **Yozbatiran et al. 2006^40^** | No | Unclear | Unclear | Unclear | 0 |
|  | | | | | |
